# Extracellular Matrix Influencing HGF/c-MET Signaling Pathway: Impact on Cancer Progression

**DOI:** 10.3390/ijms19113300

**Published:** 2018-10-24

**Authors:** Heydi Noriega-Guerra, Vanessa Morais Freitas

**Affiliations:** Departamento de Biologia Celular e do Desenvolvimento, Instituto de Ciências Biomédicas, Universidade de São Paulo, Av. Prof. Lineu Prestes 1524, Prédio I, sala 428, 05508-000, São Paulo, SP, Brazil; hnoriegag@gmail.com

**Keywords:** c-MET, HGF, extracellular matrix, tumor microenvironment, cancer progression

## Abstract

The extracellular matrix (ECM) is a crucial component of the tumor microenvironment involved in numerous cellular processes that contribute to cancer progression. It is acknowledged that tumor–stromal cell communication is driven by a complex and dynamic network of cytokines, growth factors and proteases. Thus, the ECM works as a reservoir for bioactive molecules that modulate tumor cell behavior. The hepatocyte growth factor (HGF) produced by tumor and stromal cells acts as a multifunctional cytokine and activates the c-MET receptor, which is expressed in different tumor cell types. The HGF/c-MET signaling pathway is associated with several cellular processes, such as proliferation, survival, motility, angiogenesis, invasion and metastasis. Moreover, c-MET activation can be promoted by several ECM components, including proteoglycans and glycoproteins that act as bridging molecules and/or signal co-receptors. In contrast, c-MET activation can be inhibited by proteoglycans, matricellular proteins and/or proteases that bind and sequester HGF away from the cell surface. Therefore, understanding the effects of ECM components on HGF and c-MET may provide opportunities for novel therapeutic strategies. Here, we give a short overview of how certain ECM components regulate the distribution and activation of HGF and c-MET.

## 1. Introduction

Cancer progression is not only defined by the numerous characteristics acquired by tumor cells but also includes the contributions of the tumor microenvironment [1,2]. The elaborate tumor microenvironment composed by tumor and stromal cells within the extracellular matrix (ECM) promotes neoplastic transformation, protects the tumor from the immune system and supports tumor growth, invasion and metastasis [3,4]. ECM consists of numerous proteins and macromolecules, including proteoglycans (PGs) and glycosaminoglycans (GAGs), acting as a biological barrier [5]. However, since intercellular communication in the tumor microenvironment is driven by a complex and dynamic network of cytokines, chemokines, growth factors and proteolytic enzymes, the ECM works as a reservoir for these bioactive molecules that influence tumor cell behavior [6,7,8,9].

Among the many bioactive molecules released by tumor and stromal cells, hepatocyte growth factor (HGF) is a crucial cytokine linked to promoting cancer progression [10,11]. The binding of HGF to its receptor tyrosine kinase c-MET triggers the activation of multiple downstream signaling pathways, which contribute to proliferation, growth, migration, survival, angiogenesis, invasion and metastasis [12,13,14,15]. Indeed, the HGF/c-MET system plays an essential role in tumor–stromal crosstalk [16].

The ECM′s role in cancer progression is still unclear. Several ECM components, particularly proteoglycans and glycoproteins, act as signal co-receptors and/or bridging molecules, promoting c-MET activation. The ECM also initiates signaling events through the release of its functional fragments processed by proteolytic enzymes such as matrix metalloproteinases (MMPs). On the other hand, some components of the ECM selectively bind to various growth factors and, consequently, affect the activation of signaling pathways involved in cancer evolution [17,18,19]. This review focuses on understanding how the ECM can influence the HGF/c-MET signaling pathway during cancer progression. We emphasize the effect of specific ECM components on regulating the distribution and activation of HGF and c-MET.

## 2. Extracellular Matrix as a Reservoir for Bioactive Molecules

The ECM represents a complex three-dimensional network composed of fibrous (collagens and elastin) and adhesive proteins (fibronectin, laminin, thrombospondin, among others) that are embedded in a hydrated ground substance of GAGs and PGs [5,20]. In the tumor microenvironment, ECM components are not only synthesized and secreted by stromal cells, such as cancer-associated fibroblasts (CAFs) [2,21], but also by tumor cells [22]. 

As is well known, the ECM function as an anchorage site, barrier or movement track, hence playing both a negative and positive role in cancer progression [23]. Furthermore, the altered ECM serves as a reservoir for bioactive molecules, such as cytokines, growth factors, proteolytic enzymes and others [6,7,8]. Included among the bioactive molecules released and stored in the ECM are vascular endothelial growth factor (VEGF), hepatocyte growth factor (HGF), tumor necrosis factor alpha (TNF-α), interleukin 7 (IL-7), insulin-like growth factor 1 (IGF-1), transforming growth factor beta (TGF-β), basic fibroblast growth factors (bFGF), epidermal growth factor (EGF) and platelet-derived growth factor (PDGF) [18,24,25].

All of the molecules mentioned above are expressed and released by several types of tumor cells [26,27,28] and stromal cells, such as CAFs, tumor-associated macrophages (TAMs) and neutrophils (TANs) [2,9,29]. On the other hand, tumor and stromal cells also secrete proteolytic enzymes that modify the ECM, including MMPs (e.g., MMP-2, -7, -9, -13), A disintegrin and metalloproteinase (e.g., ADAM-8, -10, -12, -15, -17), and a disintegrin and metalloproteinase with thrombospondin motifs (e.g., ADAMTS-1, -8, -13) family members [29,30,31,32]. These enzymes together with cytokines and growth factors form a complex and dynamic network that controls the autocrine and paracrine communication between tumor cells and surrounding stromal cells, thus influencing the proliferation, migration, adhesion, invasion and metastasis of malignant cells [1,7,8,9,33,34,35].

## 3. Structures of Hepatocyte Growth Factor (HGF) and c-Met

Hepatocyte growth factor (HGF, a.k.a. scatter factor) is a pleiotropic cytokine initially synthesized and secreted as a biologically inactive single-chain precursor (pro-HGF) [36,37]. However, when extracellular proteases (e.g., serine proteases and MMPs) cleave the Arg494-Val495 peptide bond of pro-HGF, this biologically inactive precursor is converted into its bioactive form [38,39,40]. Mature HGF is a disulfide-linked heterodimer composed of an α- and β-chain. The α-chain contains an N-terminal hairpin loop and four kringle domains (K1-K4), while the β-chain consists of a serine protease-like domain [41,42]. Moreover, it is noteworthy that the N-terminal and the kringle one (K1) domains are responsible for the high-affinity binding of HGF to its receptor c-MET [43].

The c-MET is a receptor tyrosine kinase (RTK) encoded by the *c-met* proto-oncogene. c-MET is produced as a single-chain precursor and processed to the mature form by post-translational modifications [39,44]. Mature c-MET consists of an extracellular α-chain and a transmembrane β-chain linked together by a disulfide bond [45]. The extracellular portion of c-MET consists of three domains: the N-terminal Sema domain (found in semaphorin and plexin families) that encompasses the entire α-chain and part of the β-chain; a small PSI domain (found in Plexins, Semaphorins and Integrins); and four IPT domains (found in Immunoglobulins, Plexins and Transcription factors). Intracellularly, the c-MET receptor is composed of a juxtamembrane domain containing the Y1003 residue, which is involved in the receptor’s down-regulation; a tyrosine kinase catalytic domain containing the Y1234 and Y1235 residues, which is involved in signal transduction; and a docking site for adaptor proteins containing Y1349 and Y1356 residues [42,46,47].

Physiologically, HGF and its receptor, c-MET, play an essential role in embryonic development, organ morphogenesis, wound healing and tissue repair through activation of different signaling pathways that are involved in cell proliferation, motility, survival, differentiation, scattering and morphogenesis [15,38,39,48].

## 4. HGF/c-MET Signaling Pathway Mediates Cancer Progression

The c-MET receptor interacts with HGF in a paracrine, endocrine or autocrine manner [49,50,51]. As soon as the Sema and IPT domains recognize HGF, two c-MET subunits dimerize, leading to the autophosphorylation of Y1234 and Y1235 residues present in the tyrosine kinase catalytic domain. This activation induces subsequent autophosphorylation of Y1349 and Y1356 residues, thus providing a docking site for the recruitment of adapter molecules (e.g., GAB1, GRB2, SHC, CRK, PI3K, PLCγ1, SHP2 and STAT3) responsible for downstream signaling. In this way, the HGF/c-MET pathway mediates Erk/MAPK, JNK, FAK, Akt/PKB and STAT3/5 activation [42,52,53]. Nonetheless, it is important to mention that the autophosphorylation of Y1003 residue located in the juxtamembrane domain leads to internalization and degradation of the c-MET receptor. Therefore, Y1003 residue negatively regulates c-MET signaling [54].

In malignant tumors, HGF is primarily expressed and released by surrounding stromal cells, including CAFs and TAMs [16,55]. However, HGF can also be produced by several tumor cell types and is detected in the renal cell [56], colorectal [57,58] and breast carcinomas [59,60], glioma [61], multiple myeloma [62] and synovial [63], osteo- and fibrosarcoma [38]. On the other hand, the c-MET receptor is overexpressed in several solid tumors, such as medulloblastoma [64], lymphoma [65], melanoma [66], glioma [67], breast [68], pancreatic [69], colorectal, ovarian and prostate carcinomas, as well as osteo- and some soft-tissue sarcomas [38].

Therefore, tumor and stromal cells communicate with each other through HGF, creating a microenvironment that contributes to cancer progression. For example, the HGF secreted by CAFs acts on tumor cells stimulating them not only to proliferate, invade and metastasize but also to produce a variety of HGF-inducers, such as bFGF, IL-1β, TGF-α, PDGF and prostaglandin E2 (PGE2), that act on stromal fibroblasts. Thus, the mutual interaction between tumor and stromal cells mediated by tumor-derived HGF-inducers and stroma-derived HGF, stimulates tumor cell invasion and metastasis [2,16,70,71,72,73]. Similarly, the HGF produced by adipose-derived stem cells (ASCs), together with the c-MET expressed in primary breast carcinoma cells, increases tumor cell migration, metastasis and self-renewal through PI3K-mediated GS3K inactivation and β-catenin stabilization and nuclear accumulation [74].

On the other hand, it was shown that c-MET aberrant activation can promote glioma cell survival via PI3-kinase/Akt signaling [75], squamous cell carcinoma invasion via STAT3 signaling [76], lymphoma cell adhesion via PI3K signaling [77], head and neck squamous cell carcinoma (HNSCC) proliferation via MAPK signaling [78], gastric cancer growth via Akt and Erk signaling [79], prostate cancer EMT and invasion via Erk/MAPK signaling [80], and breast carcinoma cell motility and lung cancer invasion via FAK signaling [81,82]. Importantly, HGF-induced tumor cell motility and invasion are accompanied by an increase in cell dissociation and protease production [e.g., MMP-2 and urokinase-type plasminogen activator (uPA)] [38,47]. Moreover, HGF/c-MET axis can stimulate the metastatic spread of colorectal tumor cells via WNT signaling [83], prostate tumor cells through induction of c-Src activity [84] and HNSCC via Erk and Akt signaling [85]. It was observed that in esophageal squamous cell carcinoma (ESCC), HGF-induced angiogenesis is driven by either pro-angiogenic cytokines (e.g., VEGF and IL-8) [86,87] or independently of VEGF, through Akt and Erk activation in endothelial cells [88]. Thus, the HGF/c-MET signaling pathway induces different phenotypes and enhances the aggressive nature of tumor cells during cancer progression.

## 5. Extracellular Matrix (ECM) Interferes with HGF/c-MET Signaling Pathway Activation

The ECM plays a critical role during cancer progression. Many ECM proteins are involved in the positive regulation of HGF/c-MET signaling. For instance, several proteases like HGFA, matriptase and MMP-2 participate in the release of mature-form HGF [89,90]. Also, MMP-1, ADAM-17 and ADAMTS-1 have “sheddase” activity and are capable of releasing membrane-bound epidermal growth factor (EGF)-like growth factors, including transforming growth factor alpha (TGF-α) [91,92,93,94]. Once EGFR is activated by TGF-α or EGF, it interacts with c-MET [95]. Such crosstalk induces ligand-free activation of c-MET signaling and leads to increased tumor cell proliferation, survival, migration, invasion, angiogenesis, and metastasis [96,97].

Some ECM components such as cell surface heparan sulfate proteoglycans (HSPGs) can bind to different growth factors and function as signal co-receptors or presenters. Indeed, CD44 and syndecan-1 can interact and recruit HGF on the cell membrane, thereby facilitating the presentation of this ligand to c-MET [17,98,99,100]. CD44 also interacts with c-MET and improves tumor cell migration, invasion and metastasis [47]. Moreover, α5β1 and α6β4 integrins directly bind to c-MET and enhance its signaling. In addition, glycoproteins including laminin and fibronectin, have the ability to promote the association between c-MET and integrin α5β1 or α6β4, leading not only to ligand-free activation of c-MET but also to amplification of signaling by both receptors [101,102,103]. It was shown that c-MET displaces α5 integrin from β1 integrin, forming a powerful structural c-Met/β1 complex that exhibits a greater affinity for fibronectin during the invasive and metastatic process [104].

On the other hand, some proteoglycans can interact with a large number of macromolecules (e.g., growth factors and water molecules) [105]. In general, proteoglycans consist of one or more sulfated GAG chains covalently attached to a core protein [106,107]. All GAGs (except hyaluronic acid) contain sulfate groups that render the proteoglycans negatively charged. In this way, the proteoglycan through its sulfated GAGs chains can attract positively charged growth factors [105,108]. It was shown that HGF binds with high affinity to heparin, specifically with *N*-sulfate, 2-*O*-sulfate, and 6-*O*-sulfate groups [109,110]. Also, heparin has the ability to displace HGF from the HSPGs, suggesting that cell surface HSPGs present low-affinity binding sites to HGF. Specifically, the minimal heparin/heparan sulfate size for binding to HGF is an octasaccharide [111,112]. 

Cell-surface HSPGs act as signal co-receptors or presenters for c-MET, thus playing an important role in normal biological functions. The proteoglycans not only regulate the delivery of HGF to the target cells but also control the activity of HGF [112]. The heparin on the ECM acts as a reservoir and impairs the interaction of HGF with c-MET, leading to reduced mitogenic and motogenic responses of cells to HGF [111,113,114,115]. However, recent studies show that heparin can induce c-MET activation in the absence of HGF. Indeed, heparin promotes tumor-cell motility and invasion through activation of c-MET signaling pathway [116,117,118].

Another ECM component that is capable of sequestering HGF is the thrombospondin-1 (TSP-1), a matricellular protein involved in angiogenesis, inflammation and cancer [119]. TSP-1 binds to HGF likely through its heparin-binding domains and mobilizes HGF away from the cell surface HSPGs, thus preventing c-MET activation [120,121]. Recently, we have shown that HGF/c-MET signaling pathway can also be affected by ADAMTS-1 through a mechanism speculated to involve degradation of HGF by ADAMTS-1 or its sequestration by ADAMTS-1 TSP-1 motifs [122]. However, growth factors attached to ECM components can be released by MMPs and ADAMTSs and thus activate different signaling pathways involved in cancer progression [94].

## 6. Strategies to Target HGF/cMET–ECM Interactions

An understanding of the effects of ECM components on HGF and c-MET pathway has provided ideas to therapeutic strategies. Initially, heparin sulfate-binding peptides were designed to block interactions with its ligands, hence disturbing involved signaling pathways [123,124]. Inhibiting heparan sulfate proteoglycan synthesis or altering heparin posttranslational modifications were also considered. The sulfatase HSulf-1 is responsible for modulating heparin-binding growth factors, including HGF. It is involved in the sulfation of specific sites of heparan sulfate glycosaminoglycans (HSGAGs), which is critical for HGF/HS interaction. Also, HSulf-1 was described as a negative regulator of HGF interaction with c-MET [125]. Another idea is based on the fact that HGF basic residues comprise the primary heparan sulfate (HS) binding sites. The substitution of residues on HGF sequence transformes it into a selective competitive antagonist, with the ability to block the c-MET pathway [126]. Concerning HGF/c-MET specific drugs, two major classes are described: the small molecules tyrosine kinase inhibitors (TKIs), whose mechanism of action is mainly intracellular and the human monoclonal antibodies that recognize extracellular components. A comprehensive review of these drugs was performed by De Silva et al. (2017) [127]. Monoclonal antibodies targeting HGF/c-MET pathways have been evaluated in clinical trials and are able to recognize the HGF or the c-MET extracellular domains, ultimately disturbing HGF/c-MET interaction and signaling. It has been described that the tumor-associated extracellular matrix (TAECM) masks epitopes and becomes an impediment for monoclonal antibody therapy, since accumulation of hyaluronan (HA) hampers tumor infiltration by natural killer (NK) cells and consequent tumor cell-mediated cytolysis. In addition, the use of PH20 hyaluronidase depletes tumoral HA and results in an increase of tumor cell death in the presence of trastuzumab [128]. In this way, hyaluronidase could likely be used in combination with monoclonal antibodies that target HGF/c-MET components to increase success in disturbing this signaling pathway.

## 7. Conclusions

**Figure 1:**
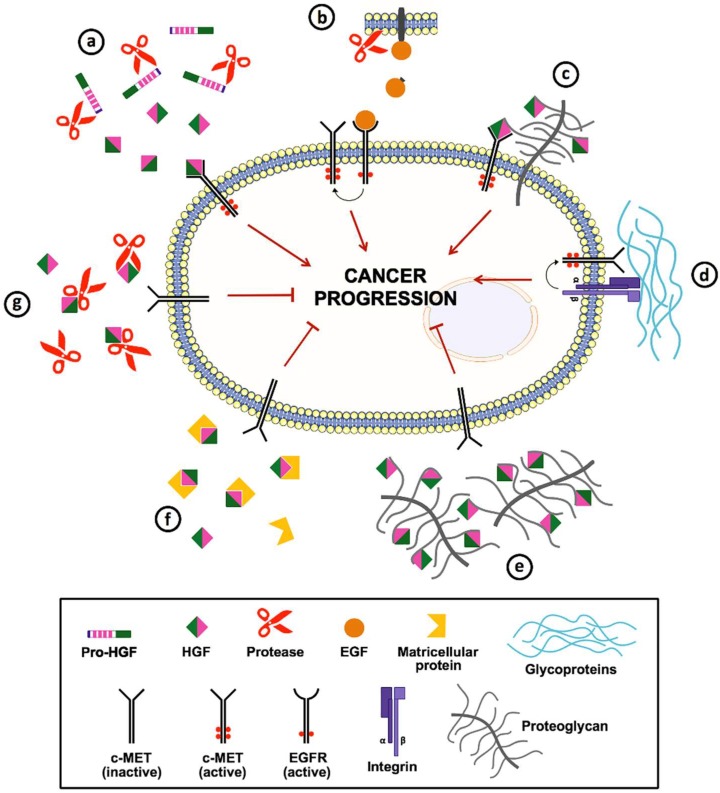
Schematic representation of the extracellular matrix (ECM) components implicated in the regulation of c-MET signaling. (**a**) Extracellular proteases (e.g., HGFA, matriptase and MMP-2) are able to cleave and convert pro-hepatocyte growth factor (HGF) (single-chain inactive precursor) into its biologically active form. (**b**) Besides, some proteases (e.g., MMP-1, ADAM-17 and ADAMTS-1) mediate the shedding of EGFR ligands from the cell membrane; the activated EGFR can interact with c-MET (crosstalk), resulting in the latter’s ligand-free activation. (**c**) Cell surface heparan sulfate proteoglycans (e.g., CD44 and syndecan-1) bind to HGF and act as co-receptors and presenters for c-MET. (**d**) Glycoproteins (e.g., laminin and fibronectin) promote the association of integrins with c-MET, leading not only to ligand-free activation of c-MET but also to amplification of signaling by both receptors. (**e**) In contrast, some proteoglycans (e.g., heparin) can directly bind to HGF and prevent its interaction with c-MET. (**f**) Matricellular proteins (e.g., TSP-1) are another ECM components capable of sequestering HGF. (**g**) Also, specific proteases (e.g., ADAMTS-1) can sequester or degrade HGF, preventing the activation of HGF/c-MET signaling pathway.

The ECM works as a reservoir for bioactive molecules, including cytokines, growth factors and proteases that modulate tumor cell behavior. HGF produced by tumor and stromal cells acts as a multifunctional cytokine that plays an important role in cancer progression by promoting proliferation, survival, motility, angiogenesis, invasion and metastasis. However, this signaling pathway can be influenced directly or indirectly by ECM. Figure 1 summarizes the roles of several ECM components in the regulation of c-MET signaling. Among these, proteases play an essential role not only in HGF activation but also in the shedding of HB-EGF and TGF-α, which induce the ligand-free activation of c-MET through its interaction with the activated EGFR. In addition, proteoglycans and glycoproteins can act as signal co-receptors or bridging molecules and activate c-MET. However, this activation can be disturbed by some ECM components, including matricellular proteins, proteoglycans and proteases, which bind and sequester HGF away from c-MET. Consequently, ECM components that interact with HGF and c-MET can regulate the distribution and activation of these two molecules, leading to either progression or inhibition of tumorigenesis.

## Abbreviations

ADAM	A disintegrin and metalloproteinase
ADAMTS	A disintegrin and metalloproteinase with thrombospondin motifs
ADSCs	Adipose-derived stem cells
bFGF	Basic fibroblast growth factors
CAFs	Cancer-associated fibroblasts
ECM	Extracellular matrix
EGF	Epidermal growth factor
EGFR	Epidermal growth factor receptor
EMT	Epithelial mesenchymal transition
ESCC	Esophageal squamous cell carcinoma
GAGs	Glycosaminoglycans
HB-EGF	Heparin-binding epidermal growth factor
HGF	Hepatocyte growth factor
HS	Heparan sulfate
HNSCC	Head and neck squamous cell carcinoma
HSPGs	Heparan sulfate proteoglycans
IGF-1	Insulin-like growth factor 1
IL	interleukin
MMPs	Matrix metalloproteases
NK	Natural killer
PDGF	Platelet-derived growth factor
PGs	Proteoglycans
PG2E	Prostaglandin E2
RTK	Receptor tyrosine kinase
TAMs	Tumor-associated macrophages
TANs	Tumor-associated neutrophils
TGF-α	Transforming growth factor alpha
TGF-β	Transforming growth factor beta
TKIs	Tyrosine kinase inhibitors
TNF-α	Tumor necrosis factor alpha
TSP	Thrombospondin
TAECM	Tumor-associated extracellular matrix
uPA	Urokinase-type plasminogen activator
VEGF	Vascular endothelial growth factor
